# Activation of the ABA Signal Pathway Mediated by GABA Improves the Drought Resistance of Apple Seedlings

**DOI:** 10.3390/ijms222312676

**Published:** 2021-11-24

**Authors:** Chenlu Liu, Hongtao Wang, Xiuzhi Zhang, Fengwang Ma, Tianli Guo, Cuiying Li

**Affiliations:** State Key Laboratory of Crop Stress Biology for Arid Areas/Shaanxi Key Laboratory of Apple, College of Horticulture, Northwest A&F University, Xianyang 712100, China; 2018050274lcl@nwafu.edu.cn (C.L.); wht17854273332@163.com (H.W.); zhangxiuzhi0124@163.com (X.Z.); fwm64@nwafu.edu.cn (F.M.); guo1003tianli@nwafu.edu.cn (T.G.)

**Keywords:** exogenous, GABA, antioxidant enzymes, stomata, ABA, drought stress

## Abstract

Drought seriously affects the yield and quality of apples. γ-aminobutyric acid (GABA) plays an important role in the responses of plants to various stresses. However, the role and possible mechanism of GABA in the drought response of apple seedlings remain unknown. To explore the effect of GABA on apple seedlings under drought stress, seedlings of *Malus hupehensis* were treated with seven concentrations of GABA, and the response of seedlings under 15-day drought stress was observed. The results showed that 0.5 mM GABA was the most effective at relieving drought stress. Treatment with GABA reduced the relative electrical conductivity and MDA content of leaves induced by drought stress and significantly increased the relative water content of leaves. Exogenous GABA significantly decreased the stomatal conductance and intercellular carbon dioxide concentration and transpiration rate, and it significantly increased the photosynthetic rate under drought. GABA also reduced the accumulation of superoxide anions and hydrogen peroxide in leaf tissues under drought and increased the activities of POD, SOD, and CAT and the content of GABA. Exogenous treatment with GABA acted through the accumulation of abscisic acid (ABA) in the leaves to significantly decrease stomatal conductance and increase the stomatal closure rate, and the levels of expression of ABA-related genes *PYL4*, *ABI1*, *ABI2*, *HAB1*, *ABF3*, and *OST1* changed in response to drought. Taken together, exogenous GABA can enhance the drought tolerance of apple seedlings.

## 1. Introduction

Actively growing plants are usually faced with various unsuitable conditions, such as drought, salt, alkaline, and nutritional stresses [1,2,3]. Among these conditions, drought stress has the most serious effect on plant growth [4]. It has been demonstrated that terrestrial plants in approximately 40% of the world’s arable land suffer from a water deficit [5]. In drought-affected areas, inadequate water use for agriculture is lead to a significant decline in crop yield and quality. Apples are one of the most important fruit crops in the world. However, the main limiting factor of apple production is water deficit. Increasing durations of drought have been reported to induce a series of physiological and biochemical changes, including a reduction in the germination rate and number of seedlings [6], stomatal closure [7], a reduction in the amino acid content and activity of antioxidant enzymes [8], and decreased leaf relative water content (RWC) and cell membrane stability (CMS). Plants adapt to arid environments by adjusting their stomatal conductance to change the transpiration rate and water use efficiency. Moreover, the relationship between stomatal function and plant water status is very complex and involves many factors [9,10,11].

The plant hormone abscisic acid (ABA) plays an important role in plant growth. ABA has been proven to be the main chemical signal substance that regulates stomatal opening and development [12]. The ABA signaling pathway contains four core components: the ABA receptor PYR/PYL/RCAR [13], protein phosphatase PP2C [14], SNF1-associated kinase 2 [15], and transcription factor ABF/AREB [16]. In the absence of ABA, PP2C inactivates SnRK2 by direct dephosphorylation, so that it cannot activate the downstream transcription factor ABF/AREB. Thus, the ABA signaling pathway cannot be activated. When plants produce a large amount of ABA under drought stress, PYR/PYL/RCAR binds with ABA to form a complex and interacts with the PP2Cs to inhibit their activity. This interaction depends on serine-threonine kinase OPEN STOMATA 1 (OST1) [17], which activates Snrk2 and then dephosphorylates the downstream transcription factor AREB/ABF to activate the ABA signaling pathway [18,19]. OST1 encodes SNF1-associated protein kinase 2 (SnRK2), which participates in ABA-mediated stomatal closure [20].

γ-aminobutyric acid (GABA), a non-protein amino acid, was first discovered in potato tubers half a century ago [21] as a signal substance or metabolite [22,23]. GABA is a four-carbon, non-protein amino acid that exists in virtually all known prokaryotes and eukaryotes. GABA is considered a significant metabolite and signal, and its biosynthesis and roles in plants have been proved [23]. GABA is synthesized in the cytosol by glutamate decarboxylase (GAD) in plants. Extensive research in the past demonstrated that abiotic stresses, such as high temperatures [24,25], salinity [26,27], osmotic stress [28], and iron deficiency [1], can lead to the accumulation of GABA in plant cells. Recent studies provided evidence of the beneficial effects of γ-aminobutyric acid (GABA) on abiotic stress in plants: Exogenously applied GABA can effectively enhance the adaptation of different plant species to drought, high temperature, salt stress, and alkaline stress [29,30]. The application of GABA particularly regulated glutathione metabolism, carbon fixation, and amino acid metabolism related to better thermotolerance [31]. GABA caused further enhancements in antioxidant enzyme activity and superoxide dismutase activities when plants were subjected to Cd [32]. GABA pretreatment alleviated iron deficiency-induced chlorosis and growth inhibition and effectively increased iron content roots [1]. As an exogenous substance, GABA has been proven to improve the tolerance of many plants under stress, but whether GABA can improve the drought tolerance of apple seedlings and how it is regulated have not been clearly elucidated.

Although it is well-known that GABA is implicated in plant tolerance and it plays a role as a signaling molecule, its effect on phytohormones is not clear. Previous results indicated that GABA works in harmony with phytohormones and suggested that regulation of phytohormones by exogenous GABA could be important in reducing plant stress [33], and exogenous GABA may be involved in ABA signaling pathways under salt stress [34]. However, there is little information about effects of GABA on plant hormones in apple seedlings under drought stress.

The objectives of this study were (1) to investigate whether GABA could enhance drought tolerance in apple (*Malus hupehensis*) through the modulation of physiological and biochemical pathways and (2) to determine the mechanism of exogenous GABA in the improvement of drought tolerance.

## 2. Results

### 2.1. Screening for the Optimal Concentration of GABA

**Figure 1:**
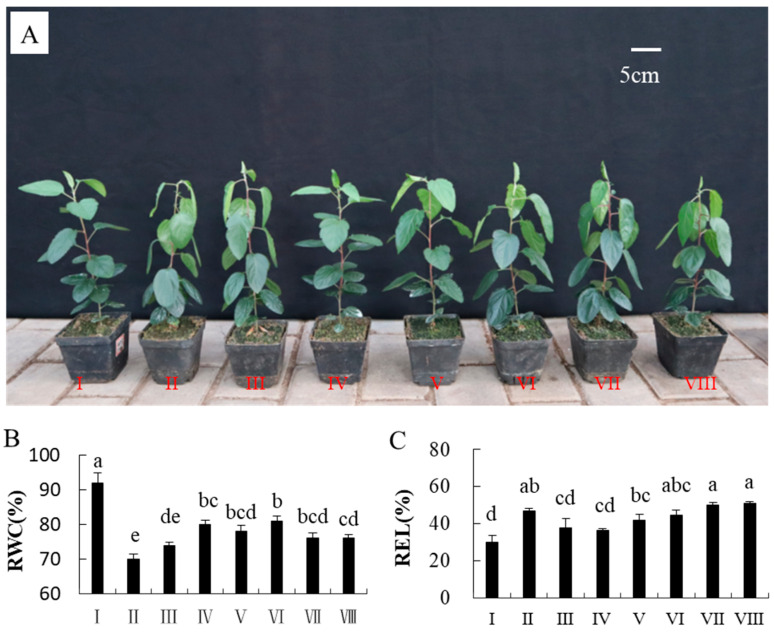
Growth status of seedlings treated with different concentrations of GABA (CK, 0, 0.25, 0.5, 1, 2, 5, and 8 mM) under drought stress (**A**). Graphical representation for the data of relative water content (RWC) (**B**) and relative electrolyte leakage (REL) (**C**) of seedlings treated with varying concentrations of GABA under drought stress. Data are the mean ± SD (n = 3). Tukey’s multiple range test (two-way) based on an analysis of variance (ANOVA) was utilized to analyze the data. Values with different letters are significantly different (*p* < 0.05).

Treatment with 0.5 mM GABA effectively improved the drought tolerance of apple seedlings (Figure 1A). Drought stress significantly decreased the RWC of leaves compared with the control. The RWC values in leaves were found to be 92%, 70%, 74%, 80%, 78%, 81%, 76%, and 76% in groups I, II, III, IV, V, VI, VII, and VIII, respectively (Figure 1B).

The values of REL in the III and IV groups were the smallest (Figure 1C). We determined that 0.5 mM is the most suitable concentration to use to alleviate drought. GABA increased the RWC and reduced the REL.

### 2.2. Plant Growth

**Figure 2:**
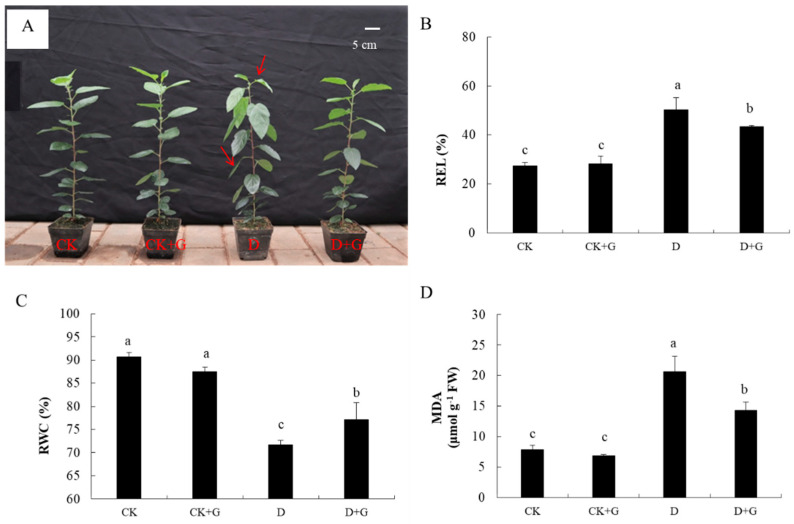
Phenotype of apple seedlings treated with γ-aminobutyric acid (GABA) application under drought stress for 15 days (**A**). CK: control; CK + G: control +0.5 mM GABA; D: drought stress; D + G: drought stress +0.5 mM GABA. The effects of GABA application on relative electrolyte leakage (REL, **B**), relative water content (RWC, **C**) and malondialdehyde content (MDA, **D**) of apple seedlings under drought stress for 15 days. Data are the mean ±SD (n = 3). Tukey’s multiple range test (two-way) based on an analysis of variance (ANOVA) was utilized to analyze the data. Values with different letters are significantly different (*p* < 0.05).

Drought stress had strong negative effects on plant growth. The leaves in the D treatments were severely wilted. However, the leaves treated with GABA still maintained good growth under drought stress (Figure 2A).

Under drought treatment, REL increased by 83.2%, and RWC decreased by 20.9% in group D compared with the control (Figure 2B,C). However, exogenous GABA significantly alleviated those decreases when compared with drought-stressed plants that had received no such supplementation. The values increased by 53.6% for REL (Figure 2B), while the degree of decreases was only 14.9% for RWC (Figure 2C) when compared with the control values; moreover, the content of MDA in D + G was 30.7% less than that in D (Figure 2D).

### 2.3. Gas Exchange Parameters

**Figure 3:**
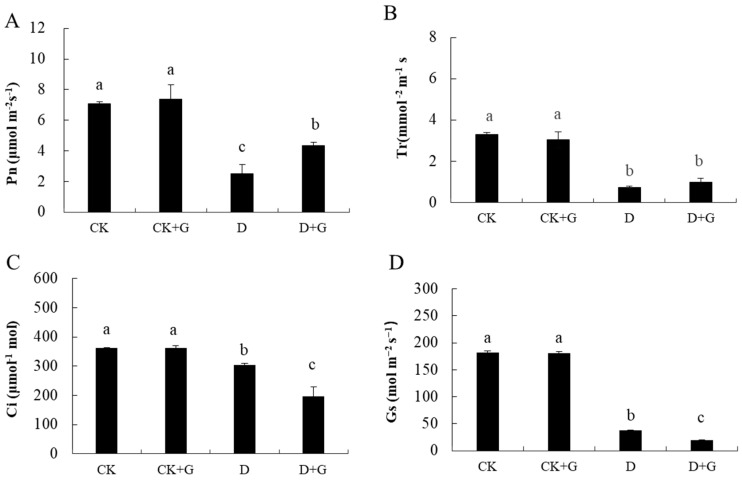
Effects of γ-aminobutyric acid (GABA) treatment on the photosynthetic rate (Pn, **A**), transpiration rate (Tr, **B**), intercellular CO_2_ concentration (Ci, **C**) and stomatal conductance (Gs, **D**) of apple seedlings subjected to drought stress for 15 days. Data are the mean ±SD (n = 3). Tukey’s multiple range test (two-way) based on an analysis of variance (ANOVA) was utilized to analyze the data. Values with different letters are significantly different (*p* < 0.05).

The values of the instantaneous gas exchange parameters measured in this study to investigate the changes in the photosynthetic capability of apple seedlings subjected to drought stress are shown in Figure 3. The highest value (7.3 μmol m^−2^ s^−1^) of Pn was found in the CK + G treatment; the value in the CK was 7.1 μmol m^−2^ s^−1^, and there was no significant difference between the CK and CK + G (Figure 3A). Compared with that in the CK, the Pn values measured under drought conditions decreased significantly by 65.3% and 38.5% in the D and D + G treatments, respectively. The Tr showed no remarkable differences among the CK and CK + G treatments, and drought stress caused a significant reduction in Tr (Figure 3B). Although the D and D + G treatments caused Ci to decrease compared with that of the CK treatment, the Ci of D treatment was 55.4% higher than that of D + G (Figure 3C). Simultaneously, the value of Gs obviously decreased under drought stress. Compared with that of the CK, the Gs of D treatment was reduced by 72.3%, and the Gs of D + G was reduced by 89.3% (Figure 3D). There was no significant difference between the CK and CK + G.

### 2.4. ROS Accumulation

**Figure 4:**
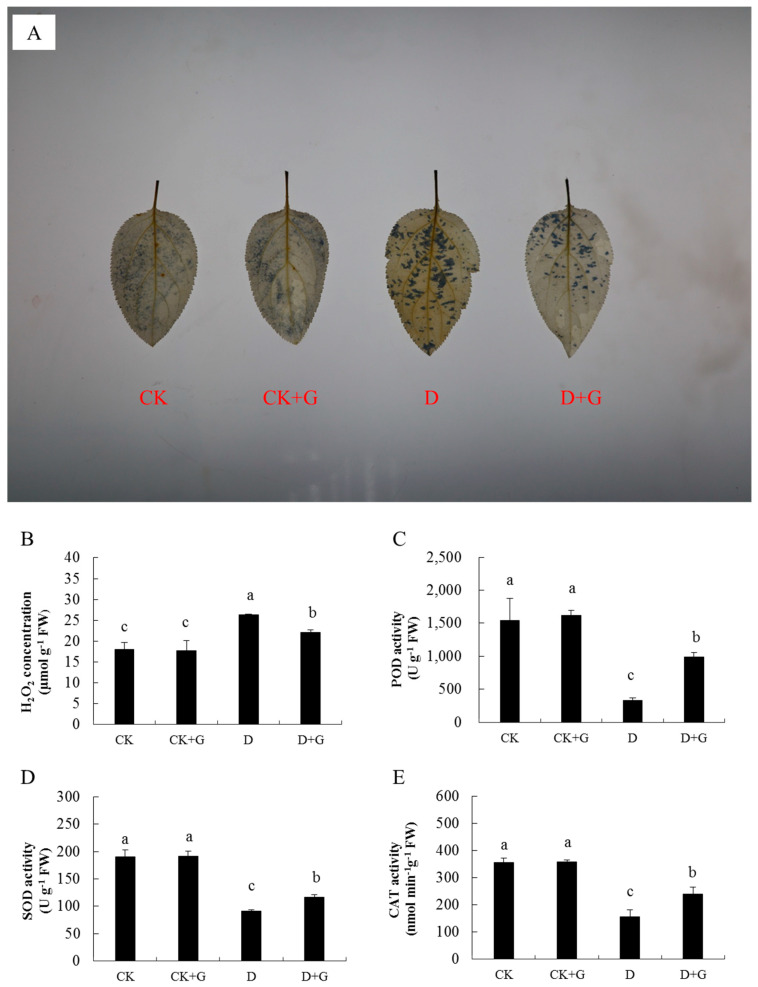
The effects of γ-aminobutyric acid (GABA) on the regulation of ROS level and antioxidant system under drought stress conditions. O_2_^−^ accumulation in detached leaves using NBT staining (**A**). Effects of GABA application on hydrogen peroxide content (H_2_O_2_, **B**), peroxidase activity (POD, **C**), superoxide dismutase activity (SOD, **D**), and catalase activity (CAT, **E**) in the leaves of apple seedlings under drought stress for 15 days. Data are the mean ± SD (n = 3). Tukey’s multiple range test (two-way) based on an analysis of variance (ANOVA) was utilized to analyze the data. Values with different letters are significantly different (*p* < 0.05).

The contents of H_2_O_2_ and O_2_^−^ and the activities of POD, SOD, and CAT were measured to reveal the accumulation of ROS in the leaves (Figure 4). The leaves of D + G treatment showed lower intensity when stained with NBT than the D leaves under drought stress (Figure 4A), indicating that D + G accumulates less O_2_^−^ than D. The CK and CK + G treatments had lower concentrations of H_2_O_2_ than the drought treatments. However, the H_2_O_2_ content of D + G was 53% less than that of D (Figure 4B).

Compared with the CK treatment, the activities of POD, SOD, and CAT decreased significantly in the D and D + G treatments. However, they were lower in D than those in D + G by 66.8%, 22%, and 34.6% respectively. There was no difference between the CK and CK + G groups (Figure 4C–E). These results revealed that the exogenous GABA reduced the accumulation of ROS under drought stress.

### 2.5. Effects of GABA on the Stomata

**Figure 5:**
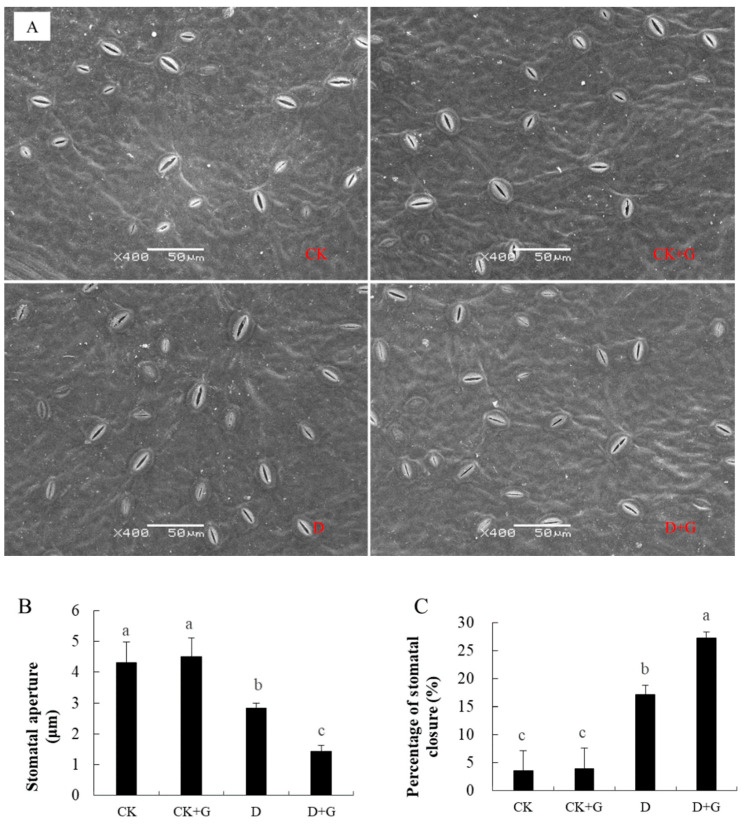
Stomatal behavior of plants after cultivation under well-watered and drought treatment conditions for 15 days. Stomatal guard cells of the plants were observed at the end of treatment via scanning electron microscopy (**A**). Stomatal aperture (**B**) and percentage of stomatal closure (**C**) were observed at the end of treatment. Data are the mean ±SD (n = 3). Tukey’s multiple range test (two-way) based on an analysis of variance (ANOVA) was utilized to analyze the data. Values with different letters are significantly different (*p* < 0.05).

Stomatal regulation is one of the important ways that plants manage a water deficit. The stomata open normally under CK and CK + G conditions (Figure 5A). The stomatal opening decreased noticeably owing to drought stress. However, the stomatal conductance of D + G was the smallest.

The stomatal aperture of D was 0.03 μm, but it was 0.014 μm in D + G (Figure 5B). The percentage of stomatal closure of D + G was higher than that of D (Figure 5C), indicating that more stomata were closed in D + G.

### 2.6. GABA and ABA Concentration and Gene Expression

**Figure 6:**
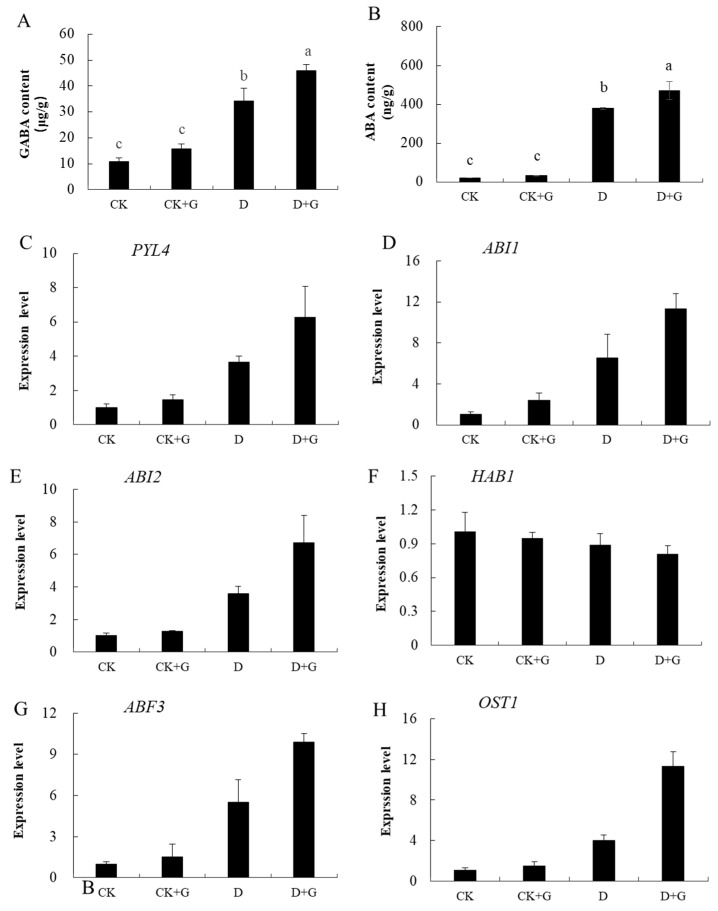

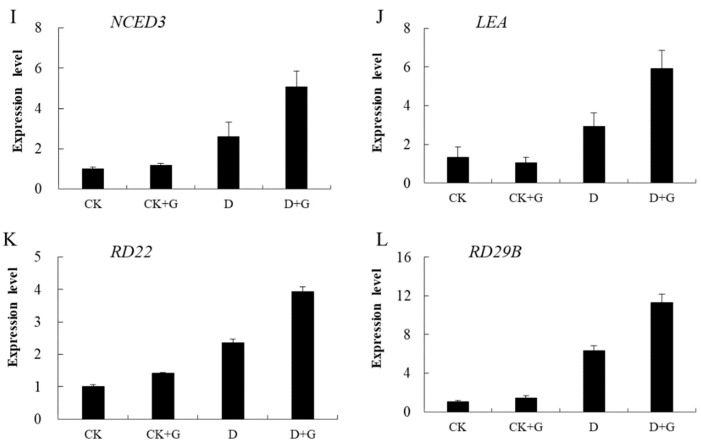
γ-aminobutyric acid (GABA) content of plants treated for 15 days (**A**). ABA content of plants treated for 15 days (**B**). Analysis of expression of ABA signaling-related genes and stress signaling genes in plants under well-watered and drought conditions (**C**–**L**). Tukey’s multiple range test (two-way) based on analysis of variance (ANOVA) was utilized to analyze the data. Values with different letters are significantly different (*p* < 0.05).

Stomatal closure is one of the most important physiological responses regulated by ABA. Under the conditions of normal watering, there was no significant difference in the content of GABA and ABA between the CK and CK + G groups. However, after 15 days of drought stress, compared with the CK, the content of GABA and ABA in D and D + G increased significantly. The content of GABA of D + G was 17.8% higher than that of D (Figure 6A), and the ABA content of D + G was 36.8% higher than that of D (Figure 6B).

We also examined the expression of genes related to the ABA signaling pathway, and the expression of these genes increased significantly under drought compared with those under sufficient water conditions (Figure 6C–E,G–I). In addition, the expression of *HAB1* decreased (Figure 6F). The results showed that compared with the CK, the levels of expression of *PYL4*, *ABI1*, *ABI2*, and *OST1* increased under drought, and the level of expression of *ABF3* in D + G was significantly higher than that in the D treatment.

In apple, some studies have also explored the involvement of these genes in stress signal transduction [35]. The expression of stress signaling genes, including *RD22*, *RD29B*, and *LEA*, were studied. The results showed that the expression levels of these genes were increased after stress treatment, and the transcriptional levels were higher in D + G (Figure 6J–L).

## 3. Discussion

Under drought stress, plants usually display a decreased RWC, low turgor pressure, and a low transpiration rate [36], causing lipid peroxidation and the inactivation of enzymes. Drought stress induces osmotic and oxidative stresses that perturb plant metabolism and lead to membrane damage and the accumulation of lipid peroxides [2]. Our results showed that drought stress can increase the damage to cell membranes in apple seedlings. In accordance with previous findings of *Coix lacryma-jobi* L. and tomato [37,38], our results also indicated that drought stress significantly affected the activities of POD, SOD, and CAT accompanied by a rapid increase in active oxygen metabolites. However, exogenous GABA could reduce the accumulation of ROS, and the GABA content in plants increased significantly after stress, similar to the results of another study of white clover [39]. The study on white clover showed that exogenous application of GABA effectively alleviated drought-induced damage in leaves, inducing significantly higher relative water content, lower electrolyte leakage, lipid peroxidation, and leaf wilt. These results indicate the positive function of GABA in drought stress. Furthermore, the transcript levels of some drought-responsive genes, i.e., *RD22*, *LEA*, *RD29B*, and *NCED3*, were up-regulated by the addition of GABA. Those results are consistent with previous results, which showed that representative genes related to drought were up-regulated in Malus in response to drought stress are beneficial to stimulation of the anti-stress system, and enhance the stress tolerance in apple [35,40,41].

Previous findings had implicated that GABA has a positive effect of enhancing plant tolerance to both abiotic and biotic stresses. As a signal substance or metabolite, GABA has an important effect on plant growth and abiotic stress resistance by regulating the pH of cytoplasm, serving as a temporary nitrogen pool, and inducing antioxidant responses [42]. Previous studies showed that 2 mg L-1 GABA effectively alleviated drought and heat-induced stress in sunflower [43], and 0.5 mM GABA exhibited a positive function in alleviating heat-induced chlorophyll loss and photoinhibition [24]. Some protective effects of GABA on plants have also been reported for maize plants [32], tomato (Solanum lycopersicum L.) [38], and creeping bentgrass [24]. Similar results have been obtained with drought-stressed white clover (Trifolium repens), in which the application of GABA reduced lipid peroxidation and increased the relative water content, thereby reversing the adverse effects of a water deficit [39]. In our current study, physiological analyses indicated that GABA at the concentration of 0.5 mM could efficiently alleviate the REL and loss of RWC in plant leaves and maintain the normal state of growth. All of these reports, along with our current results, suggest that GABA priming mitigates the damage caused by stress.

In the case of drought stress, the photosynthesis of many plants is affected [24,37,43]. In a previous study, photosynthesis depended on the gas exchange of stomata and the effective water transport of xylem [44]. These results indicate that under conditions of water deficit, plants respond to drought stress by closing their stomata, thus preventing excess water loss in the leaf tissue, similar to a recent study on olive cultivars [45]. This practice indicates that in the case of water deficit, although stomatal conductance decreases, it can maintain the initial photosynthetic rate and maintain the plant’s growth [46]. In the current study, when water was sufficient, there was little difference in the gas exchange parameters between the CK and CK + G. However, under drought stress, exogenous GABA decreased the stomatal conductance and transpiration rate of seedlings, water retention was increased, and photosynthesis was higher than that of group D. A recent study has shown that cytosolic GABA signals modulate stomatal opening, WUE, and drought resilience transduced through negative regulation of *ALMT9* activity, and GABA’s role appears to be that of fine-tuning stomatal aperture [47]. Higher levels of GABA have been associated with improved drought tolerance in many plant species, providing the available precursors for synthesis of proteins and secondary metabolites for stress defense [33,48]. We hypothesized that GABA is produced in the cytosol in times of stress, that GABA priming also induces production of GABA in plants as a plant signaling molecule, and that cytosolic GABA has a role of inhibition of stomatal closure.

Our results showed that stomatal aperture was reduced and water retention and enzyme activity were increased, maintaining a higher photosynthetic rate with GABA application. However, the increase in photosynthesis may be owing to a variety of reasons. Theoretically, at the leaf level, the carboxylation rate can be increased by increasing the concentration of catalytically active RUBISCO sites, improving the kinetic characteristics of the enzyme [49,50], or bypassing photorespiration [51]. Another option is to increase the diffusion conductance of CO_2_ from the substomatal cavity to the fixed part of mesophyll (Gm) [52,53]. However, Gm is a complex feature, involving different structural characteristics and molecular controls; therefore, it requires additional study before it can be utilized in the field.

A previous study found that when apple (Malus domestica) was under drought stress, ABA signaling transduction mediated stomatal regulation and a variety of ABA/stress-related gene expression to cope with drought conditions [54]. Under drought stress, ABA synthesis increased significantly and was transported to the leaves through the xylem and phloem, entering the guard cells so that the stomata are closed and the evaporation of water is reduced [55]. ABA-mediated stomatal closure is the foremost response of plant to drought stress [54] and activates the genes related to the induction of drought resistance [4].

Since we observed that the stomatal conductance decreased significantly after short-term drought, we measured the content of ABA in leaves to explore the reason for this decrease. Our results showed that ABA accumulates greatly in leaves, particularly in the D + G treatment (Figure 6B). The concentration of ABA increased in plant tissues to promote stomatal closure and avoid excessive water loss [56], demonstrating that GABA and ABA have a synergistic effect on stomatal closure.

The plant hormone abscisic acid (ABA) functions as a chemical signal in response to environmental stresses. When plants are subjected to stress, these stress signals are converted to ABA, and this triggers the activation of plant physiological and developmental processes, thereby inducing adaptation to the stress conditions [57,58]. Under drought stress, ABA synthesis increased significantly. ABA receptors, including PYR/PYPE, control ABA signal transduction by inhibiting PP2Cs [13,59]. PYL4 is a gene that encodes PYL receptors that have previously been shown to be responsive to drought stress [54]. In this research, the expression of these genes, ABI1, ABI2, OST1, ABF3, was induced by drought and was higher in D + G. Transcriptional levels of genes related to the ABA pathway were also consistent with the changes in ABA content. Previous findings have shown that the key molecules of PP2Cs in ABA signal transduction include *ABI1*, *ABI2*, and *HAB1* [18]. *HAB1* is a negative regulator of ABA signal transduction [60]. *ABI1* regulates stomatal opening in Arabidopsis thaliana, and the stomatal opening of an abi1-1 ineffective mutant was significantly larger than that of the wild type [61]. The *ABFs* gene is a positive regulator of leaf senescence and overall survival induced by ABA [62]. Our results suggest that that GABA can participate in the ABA signaling pathway by controlling ABA levels, increasing the strength of other stress-responsive pathways to enhance the drought tolerance. Previous reports had indicated that GABA works in harmony with phytohormones and suggested that regulation of phytohormones by exogenous GABA could play a key role in reducing plant stress [33]. GABA may act as a signaling molecule and participates in regulating the expression of many genes including hormones and stress response [63]. These results indicate that the ABA signal path was activated under drought stress, especially in plants treated with GABA. We found that, in the presence of GABA, the production of ABA was increased under drought stress. Thus, it is likely that GABA helped improve drought tolerance by increasing the content of ABA in our plants and then enhanced tolerance to drought stress.

## 4. Materials and Methods

### 4.1. Plant Material and Growth Conditions

Apple plants were cultivated under greenhouse conditions in Yangling, Shaanxi province, China. *M. hupehensis* has apomixis characteristics. Its seedlings grew consistently. In this experiment, the seeds of *M. hupehensis* were collected at Pingyi in Shandong Province, China, and treated by stratification at 4 °C for approximately 40 days. They were then planted in plates that contained identical amounts of a mixture of perlite/vermiculite/peat (1:1:6 *v*/*v*/*v*). During growth the seeds were watered every 4 days during their growth period to maintain soil moisture content at 65–75% field capacity. The light-exposure time was 14 h within a 24 h period, and the light intensity was 8000–9000 Lx. The average temperature was 26 °C. Deionized water was used to irrigate the seedlings and maintain the levels of soil moisture at 65–75% field capacity.

### 4.2. Screening for Optimal GABA Concentrations

Seven drought treatments were established to screen suitable concentrations of GABA. A total of 160 apple seedlings that were two months old were randomly divided into eight groups. The apple seedlings in group I were watered with deionized water every 4 days as the control to maintain the soil moisture content at 70–75% field capacity. The other six groups were designed as drought stressed and treated by deionized water that contained 0, 0.25, 0.5, 1, 2, 5, and 8 mM of GABA, respectively, which were groups II, III, IV, V VI, VII, and VIII. The plants treated with GABA were watered at the same time as the control. After watering twice, irrigation was withheld from them to achieve natural water loss, primarily owing to soil evaporation and plant leaf transpiration, and thus induce drought stress. For each treatment, the leaves of the third to fifth leaves from the top (three replicates) were harvested after 15 days to detect the relative water content (RWC) and relative electrolyte leakage (REL). Each experiment was repeated independently three times.

### 4.3. Drought Stress and Exogenous GABA Treatment

To clarify the effect of GABA on apple seedlings under drought, we performed additional research after screening to determine the optimal concentration. When grown to 6–8 true leaves, the seedlings were replanted into plastic pots (9.5 cm high and 10 cm in diameter), which were filled with a mixture of perlite/vermiculite/peat (1:1:6 *v*/*v*/*v*) and weighed to 500 g. After 50 days, 200 healthy seedlings were selected and divided into four groups, separately designated as CK, CK + G, D, and D + G treatments. The plants of the CK and D treatments were watered with deionized water every 4 days. Moreover, the CK + G and D + G treatments were irrigated using deionized water with 0.5 mM GABA. After the last watering, groups D and D + G were no longer watered. Each watering maintained the soil moisture at approximately 70–75% field capacity. The plant samples were harvested at 15 days after natural drought for physiological and biochemical analyses. At least 30 seedlings were harvested for each treatment. Each experiment consisted of three independent biological replicates.

### 4.4. Gas Exchange Measurements

After 15 days of drought treatments, the fifth leaf was selected to measure leaf gas exchange. The photosynthetic rate (Pn, μmol m^−2^ s^−1^), stomatal conductance (Gs, mol m^−2^ s^−1^), intercellular CO_2_ concentration (Ci, μmol mol^−1^), and transpiration rate (Tr, mmol m^−1^ s^−1^) were measured using an open gas exchange system (CIRAS-3, PP-system, Hitchin, UK) between 14:00–16:00 on a sunny day. The CO_2_ concentration and photosynthetic photon flux density in the leaf chamber were maintained at 400 ppm and 1200 mol photons m^−2^ s^−1^, respectively. Twenty plants in each treatment were selected randomly.

### 4.5. RWC, REL, and MDA of Leaves

The relative electrolyte leakage (REL) was determined as previously described [29]. The relative water content (RWC) was determined gravimetrically and calculated as follows: RWC = [(FM-DM)/(TM-DM)] ×100%. On the 15th day of drought, the fresh weight (FW) and the dry mass (DM) of leaves from each treatment were measured. The turgid mass (TM) was recorded after the fresh leaves were soaked in distilled water for 24 h in a closed container at 4 °C in the dark. Ten leaves were collected for each treatment, and this was repeated three times. The malondialdehyde (MDA) content was measured using the TBA method as previously described [64].

### 4.6. Detection of Antioxidant Enzyme Activities and NBT

On the 15th day, mature leaves were gently cleaned, soaked in NBT, placed in the dark for 4 h, and finally decolorized with 85% anhydrous ethanol. NBT staining was used to detect the content of superoxide anions (O_2_^−^). The concentration of H_2_O_2_ was detected using a plant H_2_O_2_ extraction kit (Sangon Biotech, Shanghai, China).

A total of 0.1 g of leaves was used to detect the activities of antioxidant enzymes. The activities of SOD, POD, and CAT were detected by plant SOD, POD, and CAT extraction kits (Sangon Biotech, Shanghai, China) following the manufacturer’s instructions.

### 4.7. Concentrations of GABA and ABA

GABA was extracted and measured as described by Jin et al. [65]. Liquid chromatography–mass spectrometry (LC-MS) was conducted (LC: AC, ExionLC; MS:Q-trap5500, AB Sciex, Framingham, MA, USA). ABA was extracted as described by Müller and Munné-Bosch [66]. The details are as follows: The ABA extract was configured based on the volume ratio of methanol, isopropanol, and acetic acid at 20:79:1 and precooled at −20 °C. A total of 100 mg frozen leaves was weighed, and the ABA extract was added and ground to a homogenate in a mortar and swirled for 5 min. The homogenates were centrifuged at 12,000 rpm for 24 h at 4 °C. The supernatant was carefully transferred to a clean 2 mL centrifuge tube. A volume of 500μL of extract was added to the remaining precipitates and fully swirled for 5 min. The supernatant was extracted twice, and all the supernatants were filtered with a 0.22 um filter membrane. The content of ABA was determined using an LC: AC, ExionLC; MS:Q-trap5500, AB Sciex.

### 4.8. Observation of the Stomata in the Leaves

The stomata were observed using SEM, and the samples were treated as follows: On the 15th day of treatment, 10 leaves were randomly taken, cut into a square with a side length of 4 mm, immediately placed in 4% glutaraldehyde, pumped with a vacuum pump for 30 min, placed at 4 °C for more than 6 h, and rinsed five times with 0.2M PBS buffer (pH 6.8) for 5, 10, 15, 20, and 30 min, respectively. The samples were dehydrated with 30%, 50%, and 70% ethanol for 15 min and then with 80%, 90%, and 100% ethanol for 30 min and rinsed twice with isoamyl acetate for 25 min each. The samples were dried using a Hitachi HCP-2 zero boundary point dryer (Tokyo, Japan), and gold was sprayed after drying. The samples were observed and photographed under SEM. Each treatment examined 20 fields of vision. The stomatal aperture was measured using Image J (NIH, Bethesda, MD, USA).

Percentage of stomatal closure (%) = number of stomata closed per visual field/number of stomata per visual field ×100%.

### 4.9. qRT-PCR Analysis

The total RNA of all samples was extracted using a TIANGEN Plant Total RNA isolation Kit Plus (TIANGEN Biotech Co., Ltd., Beijing, China) according to the manufacturer’s instructions. Quantitative real-time PCR (qRT-PCR) was performed on an ABI StepOnePlus real-time PCR system (Applied Biosystems, Singapore, Singapore) using a SYBR Premix Ex TaqII (TaKaRa, Kyoto, Japan). *MdActin* was used as the internal control and was simultaneously amplified [67]. The relative level of expression of these genes was calculated using the 2^−ΔΔCT^ method [68]. Three independent biological replications were performed per sample. The sequence of primers used in the experiment is shown in Table 1.

### 4.10. Statistical Analysis

All the data were statistically analyzed using Microsoft Excel 2010 (Redmond, WA, USA) and SPSS 21.0 (IBM, Inc., Armonk, NY, USA) (*p* < 0.05). Tukey’s multiple range test (two-way) based on an analysis of variance (ANOVA) was utilized to analyze the data.

## 5. Conclusions

The results showed that treatment with exogenous GABA (0.5 mM) could effectively alleviate drought stress. GABA could induce apple seedlings to accumulate a large amount of ABA to activate the ABA signal pathway, resulting in stomatal closure, which ensured that the plant leaves could reduce water loss under drought conditions. This study shows that exogenous GABA could promote stomatal closure by increasing the accumulation of ABA and the subsequent drought tolerance of apple seedlings under drought conditions.

## Figures and Tables

**Table 1:** Gene information and primers used for real-time quantitative PCR.

Primer Names	Sequences
RT-PYL4-F	CGGCGTCGTCGCAGTACCAA
RT-PYL4-R	TCCTGAGTCACGGCGGAGCA
RT-ABI1-F	GGGAGGAACAACAAGGGA
RT-ABI1-R	AAGAAATGAACGGGTGAGAT
RT-ABI2-F	GACGACGAATGCCTAATT
RT-ABI2-R	TCTTGTGCCAGAGGAGTA
RT-HAB1-F	ACCCACCTAACCAGTCAC
RT-HAB1-R	ACCATAATCCCATCACCT
RT-OST1-F	AGCACCTGAAGTCCTATC
RT-OST1-R	ACTAAGAATCCGCCCAAT
RT-ABF3-F	AATGCTCAGTTGGGTAGTCC
RT-ABF3-R	TTCGCAGGTGAAGGCGTC
RT-NCED3-F	GCAGGAGATGATCGGCG
RT-NCED3-R	CAGAAGCAGTCGGGGCAGT
RT-RD22-F	GACATGCGTCCTGGAACAAC
RT-RD22-R	ATTTCTGGCAGCTTGTTGG
RT-RD29B-F	TGTGACAGGCGGTGAAGAAAT
RT-RD29B-R	TCAGCGATAGCGGAAGTGG
RT-LEA-F	TGGGGGAGATGACTTGGAG
RT-LEA-R	CTGCTTCAGGTGTAGAAGC
MdActin-F	TGACCGAATGAGCAAGGAAATTACT
MdActin-R	TACTCAGCTTTGGCAATCCACATC

## Abbreviations

GABA	γ-aminobutyric acid
ABA	abscisic acid
WUE	water use efficiency
SOD	superoxide dismutase
POD	peroxidase
CAT	catalase
MDA	malondialdehyde
RWC	relative water content
REL	relative electrolyte leakage
